# Alternative routes to the cell surface underpin insulin-regulated membrane trafficking of GLUT4

**DOI:** 10.1242/jcs.166561

**Published:** 2015-07-15

**Authors:** Dimitrios Kioumourtzoglou, Paul R. Pryor, Gwyn W. Gould, Nia J. Bryant

**Affiliations:** 1 Institute of Molecular, Cell and Systems Biology, College of Medical, Veterinary and Life Sciences, University of Glasgow, Glasgow, G12 8QQ, UK; 2 Department of Biology, University of York, York YO10 5DD, UK; 3 Centre for Immunology and Infection, Hull York Medical School, University of York, York YO10 5DD, UK

**Keywords:** Endosome, Gyrin, Membrane traffic

## Abstract

Insulin-stimulated delivery of glucose transporters (GLUT4, also known as SLC2A4) from specialized intracellular GLUT4 storage vesicles (GSVs) to the surface of fat and muscle cells is central to whole-body glucose regulation. This translocation and subsequent internalization of GLUT4 back into intracellular stores transits through numerous small membrane-bound compartments (internal GLUT4-containing vesicles; IGVs) including GSVs, but the function of these different compartments is not clear. Cellugyrin (also known as synaptogyrin-2) and sortilin define distinct populations of IGV; sortilin-positive IGVs represent GSVs, but the function of cellugyrin-containing IGVs is unknown. Here, we demonstrate a role for cellugyrin in intracellular sequestration of GLUT4 in HeLa cells and have used a proximity ligation assay to follow changes in pairwise associations between cellugyrin, sortilin, GLUT4 and membrane trafficking machinery following insulin-stimulation of 3T3-L1 adipoctyes. Our data suggest that insulin stimulates traffic from cellugyrin-containing to sortilin-containing membranes, and that cellugyrin-containing IGVs provide an insulin-sensitive reservoir to replenish GSVs following insulin-stimulated exocytosis of GLUT4. Furthermore, our data support the existence of a pathway from cellugyrin-containing membranes to the surface of 3T3-L1 adipocytes that bypasses GSVs under basal conditions, and that insulin diverts traffic away from this into GSVs.

## Abstract (precis)

**Summary:** Cellugyrin-containing internal GLUT4-containing vesicles provide an insulin-sensitive reservoir to replenish GLUT4 storage vesicles following insulin-stimulated exocytosis of GLUT4.

## INTRODUCTION

Insulin reduces elevated plasma glucose levels by increasing glucose transport into fat and muscle through the facilitative glucose transporter GLUT4 (also known as SLC2A4). In the absence of insulin, ∼95% of GLUT4 localises to intracellular compartments, with insulin causing redistribution to the plasma membrane (Bryant and Gould, 2011; Bryant et al., 2002). This is disrupted during the insulin-resistance underlying type-2 diabetes.

GLUT4 cycles through the surface of insulin-sensitive cells in both the presence and absence of insulin (Bryant et al., 2002; Kandror and Pilch, 2011). In the absence of insulin, GLUT4 is efficiently internalized into early or recycling endosomes from where it traffics to insulin-responsive GLUT4-storage vesicles (GSVs) (Kandror and Pilch, 2011). Insulin increases the amount of GLUT4 at the plasma membrane by dramatically increasing exocytosis from GSVs mediated, in part, by formation of a complex between the GSV-resident vesicular (v)-SNARE VAMP2 and its cognate target (t)-SNARE on the plasma membrane (Bryant and Gould, 2011; Bryant et al., 2002).

The intimate association of GSVs with the dynamic endosomal system makes their characterization challenging (Bryant et al., 2002; Kandror and Pilch, 2011). Morphological and biochemical studies have indicated that, under basal conditions, the majority of GLUT4 resides in small tubulo-vesicular structures (Bryant et al., 2002; Kandror and Pilch, 2011). It is from a subpopulation of these internal GLUT4-containing vesicular structures (IGVs) that GLUT4 mobilises to the plasma membrane in response to insulin; these by definition represent insulin-responsive GSVs (Bryant et al., 2002; Kandror and Pilch, 2011). Two populations of IGVs can be separated by density gradient fractionation (Jedrychowski et al., 2010; Kupriyanova and Kandror, 2000; Kupriyanova et al., 2002). Both contain GLUT4 and other proteins that translocate to the plasma membrane of adipocytes in an insulin-dependent manner, including VAMP2 and the insulin-responsive aminopeptidase, but can be distinguished by the presence or absence of the multi-ligand sorting receptor sortilin and the protein cellugyrin (also known as synaptogyrin-2) (Jedrychowski et al., 2010; Kupriyanova and Kandror, 2000; Kupriyanova et al., 2002). Sortilin plays a key role in GSV biogenesis (Huang et al., 2013; Shi and Kandror, 2005) and, like other GSV-residents, translocates to the plasma membrane in response to insulin, supporting the notion that sortilin-positive (cellugyrin-negative) IGVs represent GSVs (Jedrychowski et al., 2010; Kupriyanova and Kandror, 2000; Kupriyanova et al., 2002). In contrast, cellugyrin does not translocate to the plasma membrane in response to insulin and the function of cellugyrin-positive (sotillin-negative) IGVs is unknown (Jedrychowski et al., 2010; Kim and Kandror, 2012; Kupriyanova and Kandror, 2000; Kupriyanova et al., 2002; Li et al., 2009).

The data presented here are consistent with a model whereby, in addition to its well-studied role in stimulating traffic from GSVs to the cell surface, insulin regulates traffic between distinct populations of internal GLUT4-containing membranes (Xu and Kandror, 2002). We propose basal traffic from cellugyrin-positive vesicles to the plasma membrane is re-routed to sortilin-positive GSVs upon insulin-stimulation. We also demonstrate a role for cellugyrin in intracellular sequestration of GLUT4 and suggest that this provides a reservoir to replenish GSVs following stimulation with insulin.

## RESULTS AND DISCUSSION

### Insulin regulates traffic between cellugyrin- and sortilin-containing compartments

To investigate the relationship between sortilin-positive and cellugyrin-positive IGVs we used an *in situ* proximity ligation assay (PLA). The PLA uses antibody detection to determine whether proteins are in close proximity (Fredriksson et al., 2002; Söderberg et al., 2006). Secondary antibodies, with specific single-stranded oligonucleotides attached, detect primary antibodies bound to the proteins of interest (Fredriksson et al., 2002). If the proteins are in close proximity, the binding of the secondary antibodies allows hybridization of connector oligonucleotides, and enzymatic ligation then forms a circular ssDNA molecule providing a template for rolling circle amplification primed by one of the oligonucleotides from the secondary antibodies (Söderberg et al., 2006). The product of this can be detected using fluorescent oligonucleotide probes, allowing the frequency of each protein–protein association to be measured using microscopy (Söderberg et al., 2006).

We have previously used a PLA to study insulin-dependent formation of syntaxin4-containing SNARE complexes (Kioumourtzoglou et al., 2014). However, proteins do not need to be part of the same complex to give a signal, the assay simply requires that they are within close proximity, the distance reflecting the size of the antibodies and oligonucleotides used. The maximum distance between epitopes able to give a PLA signal (represented as a fluorescent dot of ∼500 nm) is estimated at 30 nm (Söderberg et al., 2006), including the size of the two antibodies (*F*_ab_=7 nm) and the coupled oligonucleotides (∼40 nucleotides) (Fredriksson et al., 2002). Given the diameter of GSVs (∼50 nm, see Bryant et al., 2002), any two proteins located on the surface of the same quarter sphere are potentially detectable by PLA. It is important to note that the fluorescent signal does not report on the localization of the associated proteins owing to the nature of the assay, but rather reports on the extent of their associations (Fredriksson et al., 2002).

**Fig. 1.**
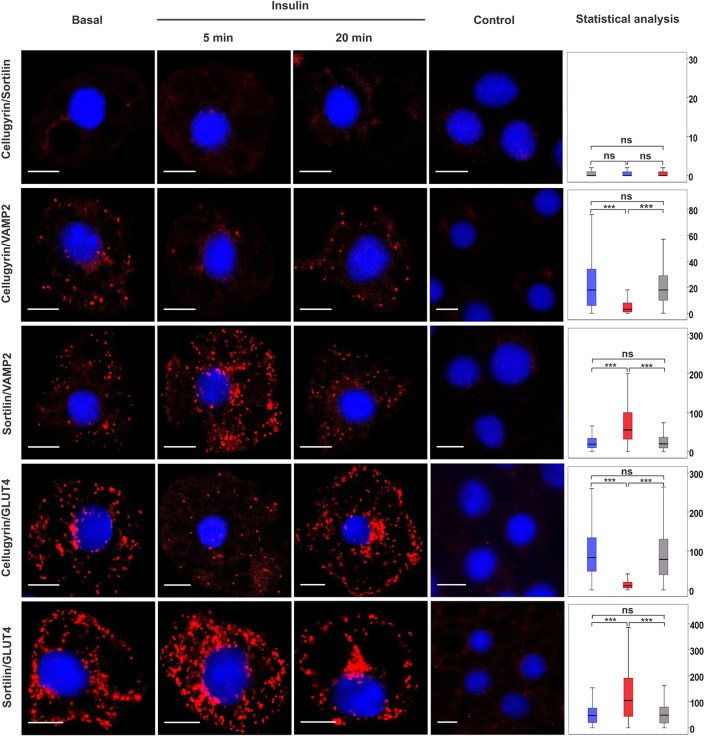
**Pairwise associations between cellugyrin, sortilin and VAMP2 or GLUT4 in the presence and absence of insulin stimulation.** A PLA was used to detect pairwise associations between cellugyrin or sortilin and VAMP2 or GLUT4 in 3T3-L1 adipocytes treated or not (Basal) with 100 nM insulin for 5 or 20 min as indicated (Insulin). PLA signals are shown in red; DAPI stain is in blue. Controls omitting the first listed primary antibody are shown (controls omitting either and both primary antibodies were performed in parallel with no significant signal detected). Statistical analyses of PLA data were performed using Blobfinder and SPSS software. The boxplots show the median number of signals and interquartile range of 200–300 cells per condition (*y*-axis: blue, basal; red, 5 min insulin stimulation; grey, 20 min insulin stimulation); the upper and the lower whiskers show the 25th and the 75th percentiles, respectively. Images are representative of three independent experiments. For all control experiments, the median PLA signal value was <1 per cell. Any median signal >1 obtained in the presence of both primary antibodies was found to be significantly greater than that obtained in controls for all combinations shown. ns, *P*≥0.05, ***P<0.001. Scale bars: 10 μm.

Consistent with fractionation studies demonstrating that sortilin and cellugyrin populate distinct populations of IGVs (Jedrychowski et al., 2010; Kupriyanova and Kandror, 2000) and the localization of VAMP2 to IGVs (Martin et al., 1997, 1996), both cellugyrin and sortilin gave a PLA signal with the v-SNARE VAMP2 but not with each other (Fig. 1). This does not provide definitive evidence that the two proteins reside in distinct IGV populations, but is noteworthy as the detection antibodies used both gave a PLA signal with VAMP2 (Fig. 1), which is found in both cellugyrin- and sortilin-positive vesicles (Jedrychowski et al., 2010; Kupriyanova and Kandror, 2000).

The number of PLA puncta is proportional to the number of associations between two proteins (Söderberg et al., 2006), but it is not possible to compare numbers of associations between different protein pairs due to variability in antibody affinity and/or avidity. Comparisons can, however, be made between changes in associations of the same pairs of proteins using the same antibodies (Kioumourtzoglou et al., 2014). At 5 min after treatment of adipocytes with insulin, a significant decrease was observed in associations of cellugyrin with both GLUT4 and VAMP2, with concomitant increases between sortilin and GLUT4 or VAMP2 (Fig. 1), consistent with the notion that acute insulin treatment stimulates GLUT4 traffic from cellugyrin- to sortilin-positive vesicles.

Although sortilin-positive GSVs are the source of GLUT4 delivered to the plasma membrane following short-term exposure to insulin (∼5 min), GLUT4 exocytosis after longer times (∼20 min) appears to involve recycling from endosomes (Xu et al., 2011; Chen et al., 2012). To investigate these temporal differences, we extended our PLA analyses to include 20 min after insulin challenge (Fig. 1). In contrast to the reduction in associations between cellugyrin and GLUT4 or VAMP2 following short-term (5 min) exposure to insulin, GLUT4–cellugyrin associations increase (Fig. 1), consistent with this set of IGVs being replenished as GLUT4 recycles from the plasma membrane (Xu et al., 2011; Chen et al., 2012). This model is also supported by the observation that cellugyrin-positive vesicles accumulate recycling proteins, for example, transferrin receptor, internalized from the cell surface (Kupriyanova et al., 2002).

### Distinct pools of internal membranes provide the source of GLUT4 traffic to the cell surface under basal and insulin-stimulated conditions

The observation that insulin stimulates translocation of sortilin, but not cellugyrin, to the plasma membrane provided the first evidence that the two biochemically identified populations of IGVs are functionally distinct (Shi and Kandror, 2005). The data in Fig. 1 build on this and suggest that cellugyrin-positive vesicles provide a reservoir for replenishment of (sortilin-positive) GSVs upon insulin-stimulation, a model consistent with studies demonstrating that, although cellugyrin does not translocate to the cell surface, GLUT4 is lost from the cellugyrin-positive compartment in response to insulin (Jedrychowski et al., 2010; Shi and Kandror, 2005).

**Fig. 2.**
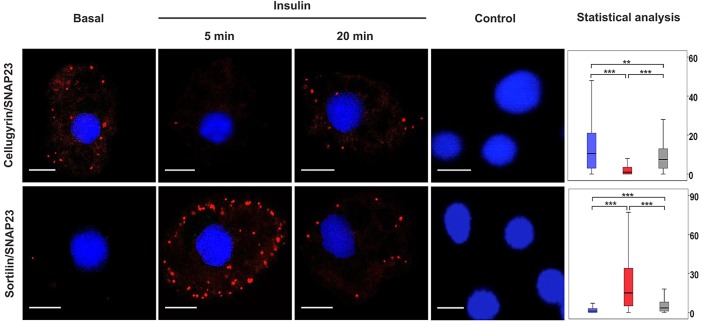
**Associations of SNAP23 with cellugyrin and sortilin in the presence and absence of insulin stimulation.** A PLA was used to detect pairwise associations of SNAP23 with either cellugyrin or sortilin in 3T3-L1 adipocytes treated or not (Basal) with 100 nM insulin for 5 or 20 min, as indicated (Insulin). PLA signals are shown in red; DAPI stain is in blue. Images are representative of three independent experiments. Data were analysed as for Fig. 1 (*y*-axis: blue, basal; red, 5 min and grey, 20 min insulin stimulation respectively). ns, *P*≥0.05, ***P<0.001, **0.001≤*P*<0.05. Scale bars: 10 μm.

To understand better how insulin-regulates traffic to the cell surface, we asked whether cargo from cellugyrin-positive internal membranes transits through sortilin-positive (cellugyrin-negative) vesicles en route to the plasma membrane under basal as well as insulin-stimulated conditions. If this were the case, cellugyrin would not encounter plasma membrane t-SNAREs. Fig. 2 shows that this is not so as cellugyrin gives a PLA signal with SNAP23 (and syntaxin4; supplementary material Fig. S2). It is important to note that although relatively few associations of cellugyrin with SNAP23 or syntaxin4 were detected per cell these represent a real signal that disappears after 5 min of insulin stimulation (Fig. 2; supplementary material Fig. S2). Consistent with reports that sortilin translocates to the plasma membrane along with other GSV residents in response to insulin but that cellugyrin does not (Kim and Kandror, 2012; Kupriyanova and Kandror, 2000), an increase in associations between sortilin and SNAP23 or syntaxin4 (Fig. 2 and supplementary material Fig. S2) was observed a concomitant reduction in the number of associations between cellugyrin and cell surface t-SNAREs at 5 min after insulin treatment (Fig. 2; supplementary material Fig. S2). This supports the existence of direct traffic from cellugyrin-positive (sortilin-negative) membranes to the plasma membrane under basal conditions and, taken in conjunction with data in Fig. 1, indicates that acute insulin challenge diverts traffic from this pathway to that mediated by sortilin-positive (cellugyrin-negative) vesicles. At longer times after insulin treatment, the cellugyrin–SNAP23 associations revert to basal levels (Fig. 2), consistent with this cellugyrin-positive set of IRVs being involved in the recycling of GLUT4 back to the plasma membrane. We speculate that this corresponds to endosomal recycling of GLUT4 as described by Xu et al. (2011) and Chen and Lippincott-Schwartz (2013), consistent with the observation that transferrin receptors recycle through cellugyrin-positive vesicles (Kupriyanova et al., 2002). Concomitant alterations in sortilin–tSNARE associations are also consistent with this (Fig. 2).

**Fig. 3.**
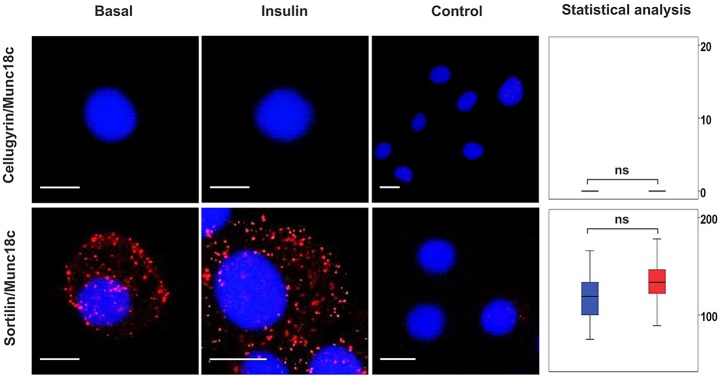
**Pairwise associations between cellugyrin, sortilin and Munc18c in the presence and absence of insulin stimulation.** A PLA was used to detect pairwise associations between cellugyrin, sortilin and Munc18c in 3T3-L1 adipocytes treated or not (Basal) with 100 nM insulin for 5 min (Insulin). PLA signals are shown in red; DAPI stain in is blue. Data were analysed and represented as for Fig. 1 (*y*-axis: blue, basal; red, 5 min and grey, 20 min insulin stimulation respectively). ns, *P*≥0.05. Scale bars: 10 μm.

Basal adipocytes contain two distinct pools of syntaxin4, one in complex with SNAP23, the other with VAMP2 and Munc18c (also known as STXBP3) (Kioumourtzoglou et al., 2014). Direct interaction between VAMP2 and syntaxin4 in the absence of SNAP23 is inhibitory to SNARE complex formation, an effect alleviated *in vitro* by a phosphomimetic (Y521E) version of the regulatory protein Munc18c but not the wild-type (Kioumourtzoglou et al., 2014). These data led to the model that the two pools of syntaxin4 are functionally distinct, that is, the pool in complex with SNAP23 facilitates basal recycling, with the other pool providing a reservoir of syntaxin4 held inactive through interaction with VAMP2, but that can be rapidly mobilized by insulin through Munc18c phosphorylation (Kioumourtzoglou et al., 2014). The simplest model integrating the data in Figs 1 and 2 into this is that recycling through the plasma membrane under basal conditions involves cellugyrin-positive (but not sortilin-positive) vesicles whereas insulin-stimulated delivery to the plasma membrane, achieved by release of syntaxin4 from the syntaxin4–VAMP2–Munc18c pool, involves sortilin-positive vesicles. Consistent with this, Munc18c is associated with sortilin vesicles, but not those marked by cellugyrin (Fig. 3).

### Cellugyrin plays a role in intracellular sequestration of GLUT4

Cellugyrin, one of four gyrin family members related to the physin and SCAMP families (Hübner et al., 2002; Kupriyanova and Kandror, 2000), is ubiquitously expressed (Hübner et al., 2002; Kupriyanova and Kandror, 2000). Little is known about cellugyrin (or any gyrin) function, but a role in vesicle biogenesis has been suggested because overexpression increases the numbers of synaptic-like microvesicles in PC12 cells (Belfort et al., 2005), and flies lacking synaptogyrin have increased synaptic vesicle diameter (Stevens et al., 2012).

**Fig. 4.**
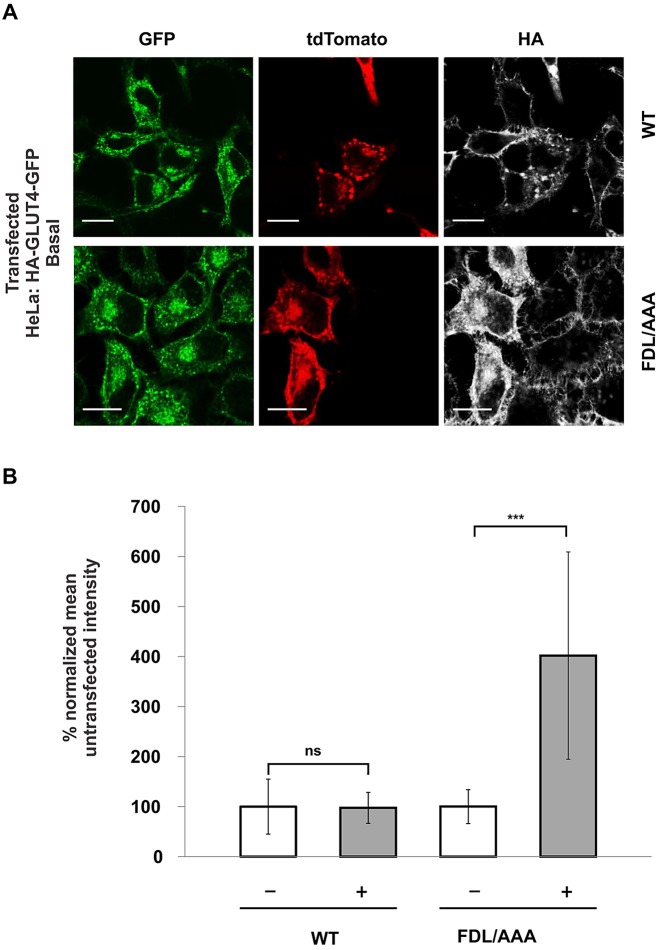
**Mutation of a short sorting sequence within the N-terminal cytosolic tail of cellugyrin (FDL to AAA) results in mislocalization to the plasma membrane and loss of intracellular sequestration of GLUT4.** A HeLa cell line stably expressing HA–GLUT4–GFP was created following infection with a lentiviral construct encoding GFP-tagged GLUT4 carrying an HA epitope in the first extracellular loop (Muretta et al., 2008). These were subsequently transiently transfected with an expression vector encoding either wild-type (WT) or mutant (FDL/AAA) tdTomato-tagged cellugyrin. (A) Indirect immunofluorescence was used to detect HA–GLUT4–GFP at the plasma membrane by staining in the absence of cell permeabilization (pseudo-coloured, white). Expression and localization of the tdTomato-tagged cellugyrin construct is shown in red and the total amount of HA–GLUT4–GFP in green. Images are representative of three independent experiments. Scale bars: 10 μm. (B) Quantification of indirect HA–GLUT4–GFP immunofluorescence (pseudo-coloured, white) at the plasma membrane was normalized to total GLUT4–GFP fluorescence from cells transfected with either WT or FDL/AAA tdTomato-tagged cellugyrin-encoding vectors (see A). The ratio of surface:total GLUT4 staining is compared between transfected (+) and non-transfected (−) cells from the same coverslip, and shows that surface levels of HA–GLUT4–GFP are increased in cells expressing FDL/AAA cellugyrin. Results represent mean±s.d. from 10 different cells (data were statistically analysed in pairs using a two-tailed Student's *t-*test; ns, *P*≥0.05; ***P<0.001.). Quantification of GFP signal from cells expressing either WT or FDL/AAA cellugyrin revealed no difference in total GLUT4–GFP levels between the two (supplementary material Fig. S3).

We have identified a short sequence within the N-terminal tail of cellugyrin similar to that required for trafficking of other endosomal proteins (Seaman, 2007). Mutation of this (FDL to AAA, denoted cellugyrin_FDL/AAA_) changed the localization of tdTomato-tagged cellugyrin from cytosolic puncta, characteristic of endosomal proteins, to the surface of HeLa cells (Fig. 4).

When HA–GLUT4–GFP (Muretta et al., 2008) is expressed in a variety of cell types, including HeLa cells, that do not normally express GLUT4, an intracellular localization with exclusion from the plasma membrane similar to that in fat and muscle cells under basal conditions is observed (Haga et al., 2011). The HA epitope is in an extracellular loop and thus only HA–GLUT4–GFP inserted into the plasma membrane is accessible to anti-HA antibody in absence of cell permeabilization. HeLa cells expressing plasma membrane localized cellugyrin_FDL/AAA_ displayed increased levels of surface HA–GLUT4–GFP (Fig. 4), demonstrating that cellugyrin localization is important for determining the localization of GLUT4. Given that cellugyrin localizes to intracellular vesicles, we propose that cellugyrin plays a role in the intracellular sequestration of GLUT4. Overexpression of either wild-type or mutant (FDL to AAA) cellugyrin did not affect the levels of HA–GLUT4–GFP in HeLa cells (supplementary material Fig. S3).

### Concluding remarks

The data presented here are consistent with a model in which, in addition to increasing delivery of GLUT4 to the plasma membrane, insulin also stimulates traffic between distinct populations of IGVs: from cellugyrin-positive to sortilin-positive vesicles. Consistent with this are reports that, unlike sortilin, cellugyrin does not translocate to the plasma membrane in response to insulin, and insulin triggers a reduction in the amount of GLUT4 in cellugyrin-positive membranes (Jedrychowski et al., 2010; Shi and Kandror, 2005). These data support a model whereby sortilin-positive membranes are the source of GLUT4 delivered to the plasma membrane in response to insulin and that cellugyrin-positive vesicles serve as a reservoir to replenish this pool. Although the PLA does not report on the dynamics of trafficking events, our demonstration of associations between cellugyrin and the cell surface t-SNAREs under basal, but not acute insulin-stimulated conditions extend this, and indicate that cellugyrin-positive membranes are the source of traffic through the plasma membrane under basal conditions (Fig. 2). Consistent with our contention that insulin diverts traffic from this route to sortilin-positive GSVs is the dramatic increase in associations of sortilin with syntaxin4 or SNAP23 in response to acute insulin challenge (Fig. 2).

Figs 1 and 2 support the existence of two functionally distinct GLUT4 trafficking pathways to the plasma membrane: from cellugyrin-positive membranes to the cell surface under basal conditions, and from cellugyrin- to sortilin-positive membranes and then to the plasma membrane following acute insulin-stimulation. The notion of distinct GLUT4 trafficking pathways under basal and insulin-stimulated conditions is supported by studies in adipocytes from transgenic mice overexpressing GLUT4, where the amount of GLUT4 at the plasma membrane under basal conditions is elevated fourfold compared to those from wild-type, but only by a factor of two following insulin stimulation (Carvalho et al., 2004). Our PLA data also reveal important differences in GLUT4 trafficking at longer times after insulin challenge. After delivery of GSVs to the plasma membrane following acute insulin treatment, GLUT4 recycles between the plasma membrane and IRVs; these different trafficking routes were identified using VAMP2–pHlorin as a reporter (Xu et al., 2011), and have been shown to involve distinct Rab proteins (Chen et al., 2013). Our data further highlight mechanistic differences between these routes to the plasma membrane, as GLUT4 recycling at longer times of insulin challenge involves cellugyrin-positive vesicles.

We recently reported the presence of two distinct pools of syntaxin4 in adipocytes under basal conditions, one in complex with SNAP23, the other with VAMP2 and Munc18c (Kioumourtzoglou et al., 2014). We suggested that the latter pool is mobilized, through Munc18c, in response to insulin. Fig. 3 shows that Munc18c does indeed participate in insulin-stimulated delivery of sortilin-membranes to the plasma membrane. Unlike its associations with the plasma membrane t-SNAREs (Fig. 2; supplementary material Fig. S1) and VAMP2 (Fig. 1), associations of sortilin with Munc18c do not increase in number in response to insulin (Fig. 3), suggesting that levels of Munc18c might be limiting for insulin-regulated traffic to the plasma membrane. This is consistent with the reported role of Munc18c phosphorylation as a regulatory switch in this process (Aran et al., 2011; Jewell et al., 2011; Kioumourtzoglou et al., 2014).

Our model predicts that a fraction of GSVs are pre-docked at the plasma membrane through syntaxin4–VAMP2 interactions (Kioumourtzoglou et al., 2014). This would allow rapid insertion of GLUT4 into the plasma membrane in response to insulin, through phosphorylation of Munc18c (Kioumourtzoglou et al., 2014). This is consistent with TIRF microscopy studies demonstrating that a large proportion of GLUT4 is within 100 nm of the plasma membrane under basal conditions in primary adipocytes (Stenkula et al., 2010), and the marked diminution of GLUT4-positive vesicles located at the periphery of 3T3-L1 adipocytes in response to insulin (Bai et al., 2007; Hatakeyama and Kanzaki, 2011), although it is important to note that the sortilin or cellugyrin status of these GLUT4-positive vesicles has not been established. After insulin stimulation, these GSVs must be replenished. We propose that cellugyrin-positive membranes provide a reservoir of GLUT4 for this. In support of this, we provide evidence for a role of cellugyrin in intracellular sequestration of GLUT4 and suggest that insulin stimulates traffic between cellugyrin-positive and sortilin-positive membranes.

## MATERIALS AND METHODS

### Proximity ligation assay

The PLA was performed using the Duolink^®^ system (Sigma-Aldrich) as described previously (Kioumourtzoglou et al., 2014) (see supplementary material Fig. S1 for further details of assay conditions and data analysis and supplementary material Table S1 for antibodies used).

### Expression constructs

tdTomato–Cellugyrin was made by excising GFP from pEGFP-C3 (NheI/EcoRI) and inserting tdTomato and rCellugyrin PCR products using In-Fusion cloning (Clontech). tdTomato-Cellugyrin_FDL/AAA_ was made using site-directed mutagenesis.

## Supplementary Material

Supplementary Material
