# Supplementary material for: Alternative routes to the cell surface underpin insulin-regulated membrane trafficking of GLUT4
Source: J Cell Sci. 2015 Jul 15;128(14):2423–9. doi: 10.1242/jcs.166561 (PMC4510850; doi:10.1242/jcs.166561)
Supplement: Supplementary Material [file supp_128_14_2423__index.html]

Supplementary Material 

# Alternative routes to the cell surface underpin insulin-regulated membrane trafficking of GLUT4

## JCS166561 Supplementary Material

- Supplementary Material
