# Supplementary material for: Alternative routes to the cell surface underpin insulin-regulated membrane trafficking of GLUT4
Source: J Cell Sci. 2015 Jul 15;128(14):2423–9. doi: 10.1242/jcs.166561 (PMC4510850; doi:10.1242/jcs.166561)
Supplement: Supplementary Material [file supp_jcs.166561_JCS166561supp.pdf]

Figure S1. *Schematic representation of Proximity Ligation Assay to examine pairwise associations between proteins in 3T3-L1 adipocytes.* PLA was performed using the Duolink® system (Sigma-Aldrich) according to the manufacturer's instructions. (1) 3T3-L1 adipocytes were grown on Labtech 8-chamber slides. Following insulin stimulation where indicated, cells were fixed with paraformaldehyde (3% [w/v]) prior to permeabilisation (0.1% [w/v] saponin) in the presence of blocking solution (2% [w/v] bovine serum albumin, 20mM glycine) for 30min. (2) Primary antibody (see Table S1 for details) incubations were performed in this BSA-GLY-SAP solution overnight at 37°C in a humidity incubator. Cells were washed using BSA-GLY-SAP prior to (3) the addition of proximity probes (provided with the kit) diluted 1/5 with BSA-GLY-SAP. Cells were incubated at 37°C in a humidity chamber for 90 min prior to washing with BSA-GLY-SAP and (4) incubation in hybridisation/ligation solution (provided by the kit) for 30 min. Cells were washed with a solution of 20mM Tris-HCl pH 7.4, containing 137mM NaCl and 0.05% [v/v] Tween-20 for 5 min prior to (5) application of the amplification/detection solution (provided with the kit) and incubation for two hours at 37°C. (6) Slides were washed thoroughly with 0.1M NaCl 0.2M in Tris-HCl pH 7.5 allowed to air dry slides and mounted with DAPI containing mounting medium (provided with the kit). PLA signals were visualized using a Zeiss LSM Pascal Exciter fluorescence system with a 100× oil immersion objective.

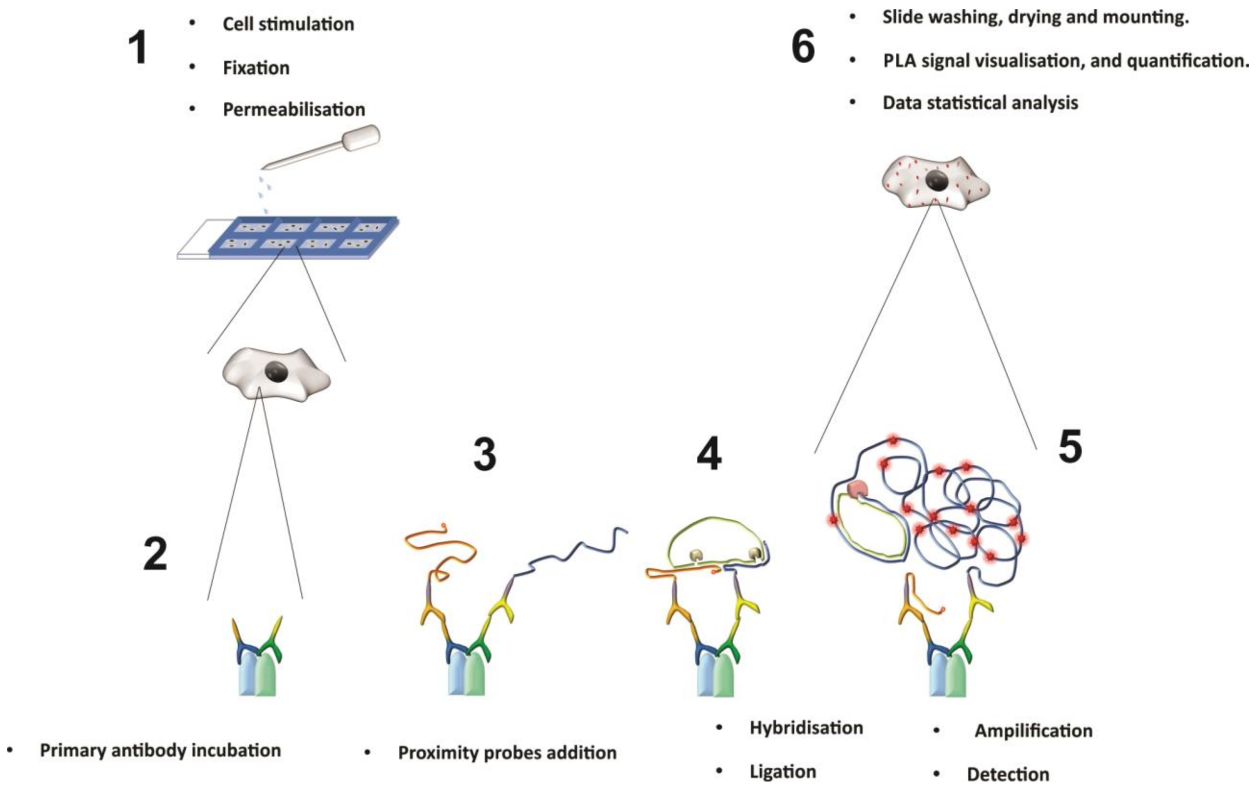

For quantification, data are presented as number of PLA puncta observed per cell (minimum of 200 cells) counted using Blobfinder version 3.2. with the following parameters used throughout: blob threshold, 120 (intensity; arbitrary units); minimum nucleus size, 100 pixels; cytoplasm size, 250 pixels; blob size, 5 by 5 pixels (i.e. in order for a puncta to be scored as a PLA signal and distinguish it from background fluorescence it must satisfy two these two parameters: blob threshold in arbitrary units and blob size in pixels, which define the minimum intensity and the minimum size respectively of a blob).

Statistical analysis of the PLA results was performed using the Mann-Whitney U test (SPSS software). Box plots presented display median values of signal per condition (from the number of cell stated, typically 200-300 cells) as well as the ranges of the values quartiles. The lower line illustrates the range of the first quartile of the sample values obtained (25% of the total number of the sample values), the box demonstrates the range of the second quartile of the values (50% of the total number) and the upper line the range of the third quartile of the values obtained. The black line in the boxes represents the median value of the samples analysed. Figures and plots are representative of the results of 3 independent experiments in all cases.

Figure S2. *Pairwise associations between Cellugyrin or Sortilin and Syntaxin4,in the presence and absence of insulin-stimulation.* PLA was used to detect pairwise associations between Cellugyrin or Sortilin and Syntaxin4 in 3T3-L1 adipocytes treated with 100nM insulin for 5 min (Insulin) or not (Basal). PLA signals are shown in red, DAPI stained nuclei in blue. Controls omitting the first listed primary antibody are shown for each pairwise combination (in all cases controls omitting either and both primary antibodies were performed in parallel and no significant signal was detected). Statistical analyses of PLA data were performed using Blobfinder and SPSS software. Boxplots represent median number of signals and quartile range of 30-50 cells per condition (y-axis; blue = basal, red =5 min insulin stimulation). Images are representative of 3 independent experiments. For all control experiments, the median PLA signal value was <1 per cell. Any median signal >1 obtained in the presence of both primary antibodies was found to be significantly greater than that obtained in controls for all combinations shown ($p < 0.001$); *** = $p < 0.001$. Scale bars = 10μm

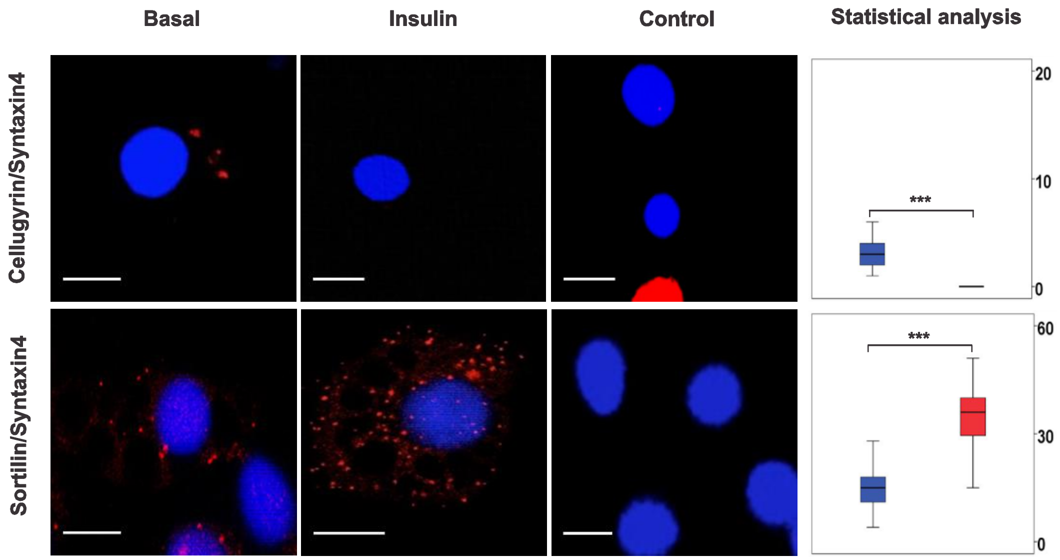

Figure S3. *Overexpression of either wild type or mutant (FDL/AAA) cellugyrin did not affect the levels of HA-GLUT4-GFP in HeLa cells.* (A) HeLa cell line stably expressing HA-GLUT4-GFP was created following infection with a lentiviral construct encoding GFP-tagged GLUT4 carrying an HA epitope in the first extracellular loop (Muretta et al., 2008). These were subsequently transiently transfected with an expression vector encoding either wild-type (WT) or mutant (FDL/AAA) tdTomato-tagged cellugyrin. Expression and localization of the tdTomato-tagged cellugyrin constructs is shown in red and the total amount of HA-GLUT4-GFP in green. Images are representative of three independent experiments. (B) Quantification of total HA-GLUT4-GFP (GFP fluorescence) of both untransfected (-) and transfected (+) cells, either with WT or FDL/AAA tdTomato-tagged cellugyrin encoding vectors, from panel A (green). The values are expressed as percentage of mean fluorescent intensity of untransfected cells. Error bars represent standard deviations from 10 different cells (data were statistically analyzed in pairs using a two-tailed t test; ns=p ≥ 0.05). Scale bars = 10μm

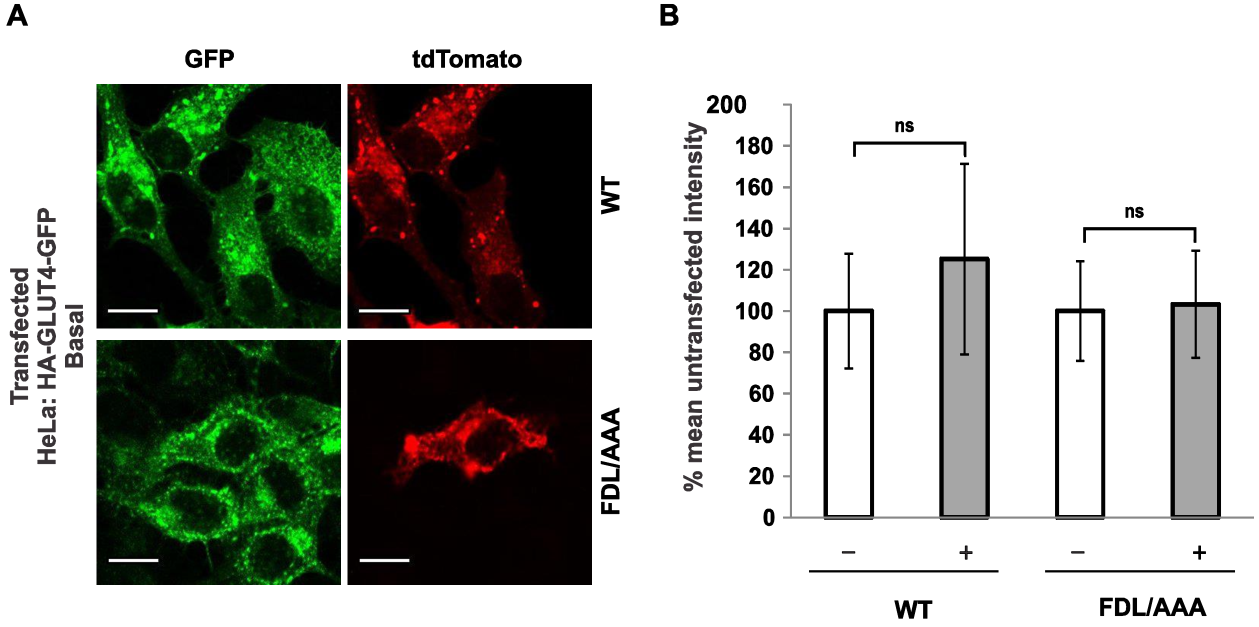

Table S1. Antibodies used in the proximity ligation assay

		Primary antibodies	
**Figure**	**PLA Protein pair associations**	**Mouse**	**Rabbit**
**1**	**Cellugyrin/Sortilin**	Anti-Cellugyrin: BD-Transduction Laboratories TM (611128); 1.25µg/ml	Anti-Sortilin: Abcam (ab16640); 5µg/ml
	**Cellugyrin/VAMP2**	Anti-Cellugyrin: BD-Transduction Laboratories TM (611128); 1.25µg/ml	Anti-VAMP2 : Abcam (ab18014); 5µg/ml
	**Sortilin/VAMP2**	Anti-VAMP2: Synaptic Systems (104211); 1:200-antiserum	Anti-Sortilin: Abcam (ab16640 ; 5µg/ml
	**Cellugyrin/GLUT4**	Anti-Cellugyrin: BD-Transduction Laboratories TM (611128); 1.25µg/ml	Anti-GLUT4: Synaptic Systems (235003); 2.5µg/ml
	**Sortilin/GLUT4**	Anti-GLUT4: Abcam (65267); 5µg/ml	Anti-Sortilin: Abcam (ab16640); 5µg/ml
**2**	**Cellugyrin/SNAP23**	Anti-Cellugyrin: BD-Transduction Laboratories TM (611128); 1.25µg/ml	Anti-SNAP23: Synaptic Systems (111203); 10µg/ml
	**Sortilin/SNAP23**	Anti-SNAP23: SantaCruz (sc-101303); 1µg/ml	Anti-Sortilin: Abcam (ab16640); 5µg/ml
**3**	**Cellugyrin/Munc18c**	Anti-Cellugyrin: BD-Transduction Laboratories TM (611128) ; 1.25µg/ml	Anti-Munc18c: Abcam (ab26331); 1:200-antiserum
	**Sortilin/Munc18c**	Anti-Munc18c: Novus Biologicals (H00006814-B01); 1:200-antiserum	Anti-Sortilin: Abcam (ab16640); 5µg/ml
**S1**	**Cellugyrin/Syntaxin4**	Anti-Cellugyrin: BD-Transduction Laboratories TM (611128) ; 1.25µg/ml	Anti-Syntaxin4: Synaptic Systems (110042); 1:200-antiserum
	**Sortilin/Syntaxin4**	Anti-Syntaxin4: BD Transduction Laboratories (610439); 1.25µg/ml	Anti-Sortilin: Abcam (ab16640); 5µg/ml
